# Use of the Exponential and Exponentiated Demand Equations to Assess the Behavioral Economics of Negative Reinforcement

**DOI:** 10.3389/fnins.2017.00077

**Published:** 2017-02-21

**Authors:** Jennifer E. C. Fragale, Kevin D. Beck, Kevin C. H. Pang

**Affiliations:** ^1^Graduate School of Biomedical Sciences, Rutgers Biomedical and Health SciencesNewark, NJ, USA; ^2^Neurobehavioral Research Lab, Department of Veteran Affairs Medical Center–New Jersey Health Care SystemEast Orange, NJ, USA; ^3^Department of Pharmacology, Physiology and Neurosciences, New Jersey Medical School—Rutgers Biomedical and Health SciencesNewark, NJ, USA

**Keywords:** behavioral economics, anxiety disorders, exponential demand equation, motivation, anxiety, depression

## Abstract

Abnormal motivation and hedonic assessment of aversive stimuli are symptoms of anxiety and depression. Symptoms influenced by motivation and anhedonia predict treatment success or resistance. Therefore, a translational approach to the study of negatively motivated behaviors is needed. We describe a novel use of behavioral economics demand curve analysis to investigate negative reinforcement in animals that separates hedonic assessment of footshock termination (i.e., relief) from motivation to escape footshock. In outbred Sprague Dawley (SD) rats, relief increased as shock intensity increased. Likewise, motivation to escape footshock increased as shock intensity increased. To demonstrate the applicability to anxiety disorders, hedonic and motivational components of negative reinforcement were investigated in anxiety vulnerable Wistar Kyoto (WKY) rats. WKY rats demonstrated increased motivation for shock cessation with no difference in relief as compared to control SD rats, consistent with a negative bias for motivation in anxiety vulnerability. Moreover, motivation was positively correlated with relief in SD, but not in WKY. This study is the first to assess the hedonic and motivational components of negative reinforcement using behavioral economic analysis. This procedure can be used to investigate positive and negative reinforcement in humans and animals to gain a better understanding of the importance of motivated behavior in stress-related disorders.

## Introduction

Abnormal motivation is associated with a number of psychiatric disorders (Robinson and Berridge, 1993; Mogg and Bradley, 1998; Berridge, 2009; Giesen et al., 2010; Treadway and Zald, 2011; Schlosser et al., 2014). Anxiety and depression demonstrate two ends of the spectrum. Individuals with anxiety disorders demonstrate enhanced motivation to avoid or escape aversive stimuli (American Psychiatric Association, 2013). On the other hand, depression is associated with impaired appreciation for reinforcement (“consummatory anhedonia”) and reduced motivation (“motivational anhedonia”) (Treadway and Zald, 2011). Symptoms strongly influenced by motivation are particularly debilitating in both conditions. In anxiety disorders, the presence of avoidance behavior is predictive of poor treatment outcomes (Foa et al., 2006; O'Donnell et al., 2007). Similarly, the presence of impaired motivation in depressed patients is also associated with poor treatment outcomes (Spijker et al., 2001) and is typically unaffected by first-line antidepressant treatment (Shelton and Tomarken, 2001). Thus, the ability to assess motivated behaviors in individuals with anxiety and depression, and to investigate motivation in pre-clinical animal models will advance the understanding of these disorders.

Aversive events play a significant role in anxiety and depression, as well as other psychiatric disorders. Stress associated with aversive events can be a precipitating event for these disorders (Kendler and Karkowski-Shuman, 1997; Kessler, 1997; Heim and Nemeroff, 2001; Nugent et al., 2011). Furthermore, anxiety is associated with negative bias, whereby people with anxiety have an enhanced attention to and motivation to remove aversive stimuli (Mogg and Bradley, 1998; Amir et al., 2003; Bar-Haim et al., 2007; Eysenck et al., 2007). In depression, the reinforcing properties of pleasurable stimuli (positive reinforcement) or the removal of aversive stimuli (negative reinforcement) are diminished, in addition to the reduced motivation to obtain both types of reinforcement (Treadway and Zald, 2011). These symptoms demonstrate the importance of studying the reinforcing properties and motivational behaviors directed at aversive stimuli. Unfortunately, no procedures are currently available that allow for the direct comparison of negative and positive reinforcement and can be readily adapted for use in both humans and animals.

Negative reinforcement (escape and avoidance) has historically been treated as a unitary process, whereas positive reinforcement with food (Hursh, 1984; Berridge, 2009) and drugs (Christensen et al., 2008b; Hursh and Silberberg, 2008; Robinson and Berridge, 2008) has been separated into hedonic and motivational components. The incentive salience theory describes independent *liking* and *wanting* components of positive reinforcement (Berridge and Robinson, 1998). Like positive reinforcement, we propose that negative reinforcement is also separable into distinct hedonic and motivational factors. Evidence from the chronic pain literature suggests that cessation of an aversive event is not only motivating, but pain relief is also rewarding (Wiech and Tracey, 2013; Navratilova and Porreca, 2014). Moreover, pain relief may use similar brain systems as positive reinforcement (Navratilova et al., 2012). Evidence suggests the nucleus accumbens (NAc) plays an important role in encoding pain relief (Navratilova and Porreca, 2014; Park et al., 2015). For example, rats exposed to a tail pinch showed suppression of extracellular dopamine in the NAc during the pinch and an increase in extracellular dopamine upon release (Park et al., 2015). Conditioned place preference (CPP) paradigms also suggest that pain-elicited motivational behavior involves dopaminergic neurons of the ventral tegmental area (VTA; Navratilova and Porreca, 2014). Despite significant overlap, recent evidence suggests that positive reinforcement and pain likely activate distinct populations of dopaminergic neurons within the mesolimbic dopaminergic system, which may modulate brain regions distinct to positive and negative reinforcement (Andreatta et al., 2012). Although the evidence is suggestive, the separation of negative reinforcement into motivation and hedonic components has not been investigated.

**Figure 1:**
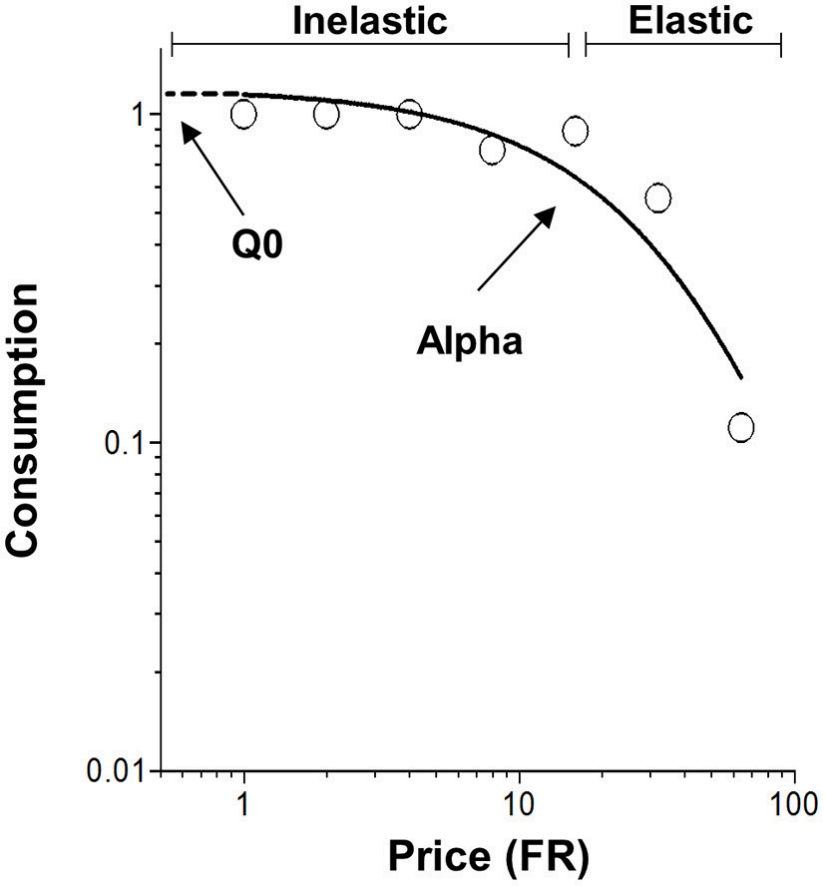
**Representative demand curve plotting consumption as a function of price (Fixed ratio, FR)**. The open circles represent successful escapes made by subjects at each FR and the solid line represents the best fit demand curve determined by the exponential demand equation. The values of *Q*_0_ and α are determined by the exponential demand equation (Equation 1). *Q*_0_ is a measure of the hedonic value of footshock and is shown graphically as the y-intercept. α is a measure of demand elasticity and is inversely related to motivation. Inelastic demand occurs when the change in consumption is small relative to the change price. However, demand becomes elastic when the change in consumption is large relative to the change price.

Similar to the incentive salience theory, independent hedonic and motivational components of reinforcement are major parts of behavioral economics analysis (Hursh and Silberberg, 2008; Bentzley et al., 2013). Behavioral economics assesses reinforcement through demand curve analysis, established by observing changes in consumption as a function of price (Hursh, 1980; Figure 1). The exponential demand equation (Equation 1) is a well-accepted demand curve analysis and has been validated in various studies using demand for actual reinforcements such as food and drugs, as well as hypothetical demand for tanning, fuel and cigarettes (Hursh and Silberberg, 2008; Bentzley et al., 2013; Reed et al., 2013, 2016; Higgins et al., 2017).

(1) $$\log_{10}Q=\log_{10}Q_{0}+k(e^{-\alpha Q_{0}C}-1)$$

In the exponential demand equation, *Q* is a measure of consumption of reinforcement. The parameter *k* is a constant that represents the range of consumption and is shared across all subjects. Cost (*C*, also called price) is the amount of work required to receive a fixed unit of reinforcement. Two important measures resulting from fitting the exponential demand equation to consumption data are (1) *Q*_0_ describing the theoretical consumption of a reinforcer when no effort is required, also used as a measure of hedonic value (Hursh and Silberberg, 2008; Bentzley et al., 2013), and (2) α a measure of demand elasticity and motivation to consume the reinforcer (Hursh and Silberberg, 2008). While these principles of behavioral economics have been widely used for positive reinforcement (Hursh, 1984; Hursh et al., 1988; Hursh and Winger, 1995; Christensen et al., 2008b; Murphy et al., 2009; Bentzley et al., 2014; Porter-Stransky et al., 2015; Rasmussen et al., 2016; Schwartz et al., 2016), they have not been applied to aversively motivated behaviors.

The present study applied behavioral economic demand analysis to negative reinforcement, specifically removal of footshock. The nature of negative reinforcement required modification of procedures previously used with positive reinforcement. Trials were used to limit the maximum number of footshocks that a rat could receive, and shock-free intertrial intervals were interspersed between trials to allow rest from shock periods. The concept of consuming reinforcement (*Q*), a mainstay of behavioral economics, is also atypical when applied to negative reinforcement, but can be simply viewed as the number of reinforcements received, similar to positive reinforcement. Whereas, *Q*_0_ is the hedonic set point for positive reinforcement (Hursh and Silberberg, 2008; Bentzley et al., 2013), *Q*_0_ serves as a measure of relief for negative reinforcement (Navratilova and Porreca, 2014). As for positive reinforcement, α is a measure of demand elasticity, describes the relationship between consumption and cost, and is inversely related to motivation for obtaining negative reinforcement.

Whereas the exponential demand equation is robust and provides a good fit for a wide variety of data, one drawback of this equation is the inability to utilize consumption values equal to 0. Because we limit the time for each trial to reduce the number of potential footshocks, consumption values of 0 may be more common than with behavioral economic procedures using positive reinforcement. Typically, values of 0 for consumption are replaced with a small number or 0 values are removed, but these methods can alter *Q*_0_ and α values (Koffarnus et al., 2015). Therefore, an exponentiated form of Equation (1) was proposed that is able to use values of zero consumption (Equation 2; Koffarnus et al., 2015).

(2) $$Q=Q_{0}\times 10^{k(e^{-\alpha Q_{0}C}-1)}$$

The present study compared the fit of the exponential and exponentiated equations to data obtained with negative reinforcement.

The procedure used in the present study has similarities to a progressive ratio (PR) task, where FRs increment in a session. PR tasks are often used to assess motivation by increasing the effort to receive reinforcement. For instance, the number of lever presses required to receive a food pellet would increase in the following manner: 1, 2, 4, 8, 16, etc. until the individual failed to lever press for a selected time period. The lever presses required at the time the rat stopped lever pressing is termed the break point and is a measure of motivation. A critical difference between the break point analysis and demand curve analysis is that the whole demand curve is analyzed in behavioral economics and only the last break point is analyzed in progressive ratio tasks. Still, we were interested in comparing a break point-like analysis to a behavioral economic analysis for negative reinforcement data.

Thus, the current study had several goals. The main goal was to determine whether behavioral economics analyses could be used to assess relief and motivation for negative reinforcement. Secondarily, we assessed the fit of two different demand equations to negative reinforcement data, and compared behavioral economics measures to breakpoint measures. Finally, we sought to determine whether behavioral economics analysis of negative reinforcement could be useful in the study of mental health disorders, specifically anxiety. The current study started by developing a novel procedure to assess hedonic and motivational components of negative reinforcement using behavioral economic analysis.

## Materials and methods

### Animals

Male Sprague Dawley (SD) and Wistar Kyoto (WKY) rats were obtained from Harlan Laboratories (Indianapolis, IN) at 3 months of age. Animals were individually housed under a 12:12 h light:dark cycle (lights on at 0700) and given *ad libitum* access to food and water. All procedures were conducted in accordance with the NIH Guide for the Care and Use of Laboratory Animals and approved by the Institutional Animal Care and Use Committee of the Veterans Affairs New Jersey Health Care System.

### Demand procedure using negative reinforcement

Rats were trained in operant chambers with grid floors for delivering scrambled footshock (Coulbourn Instruments, Whitehall, PA). The chamber walls were fit with a response lever (10.5 cm above the floor) on one side and a house light and speaker (26 cm above the floor) on the opposite side. Each operant box was housed in a sound-attenuating chamber and all chambers where controlled by Graphic State Notation software (Version 2, Coulbourn Instruments).

Rats were trained on a fixed ratio schedule requiring a single lever press (FR-1) to escape 1.0 mA footshocks for five daily sessions; each session consisted of 25 trials. At the start of each trial, rats were exposed to 1.0 mA footshocks (0.5 s duration, 3 s intershock interval, 20 shock maximum) paired with a 1 kHz tone (75 dB, continuous). A lever response immediately terminated the shock and tone, and initiated a 180 s intertrial interval (ITI). If a lever press was not made after 20 shocks, shock and tone were terminated, and the ITI was initiated. Rats that failed to respond on more than 10% of trials were removed from the study. In the present study, one SD rat was removed.

After FR-1 training, rats were tested in an ascending FR schedule. In this procedure, the number of lever responses required to terminate footshock was increased every 6 trials on a quarter logarithmic scale (the number of lever presses required increased in the following manner: 1, 2, 3, 5, 10, 18, 32). The FR schedule progressed regardless of trial performance. All other aspects of the procedure were identical to FR-1 training. To produce a stable demand curve, rats were tested for three consecutive daily sessions. To assess the effects of shock intensity, SD rats were tested at three intensities (0.5, 1.0, 2.0 mA).

### Demand procedure using positive reinforcement

In order to compare the demand characteristics for positive and negative reinforcement, a separate group of rats were trained and tested in an analogous procedure to that described for negative reinforcement. A pellet dispenser (Coulbourn Instruments, Whitehall, PA) delivered 45 mg sucrose pellets (Bioserv, Flemington, NJ) to a feeding trough located directly under the lever.

After rats were food restricted to 85% of their *ad libitum* body weight, rats were trained on an FR-1 schedule for sucrose pellets for five daily sessions (25 trials/session). A lever press resulted in the dispensing of a sucrose pellet and the termination of tone, after which rats entered a 20 s ITI. Rats were required to complete a session within 15 min to advance to the next phase. After FR-1 training, rats were tested in an ascending FR schedule with sucrose pellets substituted for escape from shock as the reinforcer.

### Exponential demand equation and curve fitting

For the present study, *Q* is consumption and operationally defined as the proportion of trials successfully completed for each FR. Mean data were obtained from three sessions for each rat. Data were fit using Equation (1) (Hursh and Silberberg, 2008). Because Equation (1) is unable to use consumption values of 0, consumption values equal to 0 were replaced with a value of 0.005, equal to 1/10 of the smallest non-zero value. Cost (*C*, or price) was the number of lever presses required to obtain reinforcement. *Q*_0_, the threoretical level of consumption when the cost approaches 0, and α, a measure of demand elasticity, were obtained from the best fit. The parameter *k* is a constant that represents the range of consumption and is shared across all subjects. A custom-designed GraphPad Prism template (Institutes for Behavioral Resources, Inc., http://ibrinc.org/software/) was used to fit the Exponential Demand Equation to the data and determine the values of *Q*_0_ and α.

### Exponentiated demand equation and data fitting

The fit of the exponentiated demand curve equation (Equation 2) was compared to that of the exponential demand equation (Equation 1). For the exponentiated demand curve fit, consumption values equal to 0 were retained, unlike the replacement of 0 values for the exponential demand equation. Values of *Q*_0_ and α were determined from the best fit of the exponentiated demand equation to the data using a GraphPad Prism template. Because our aim was to compare the best fit of the Exponential Demand Equation to that of the Exponentiated Demand Equation, the parameter *k* was not constrained to be the same for the two demand equations.

### Break point analyses

In progressive ratio procedures, break point is the FR on which animals cease to respond and is used as a measure of motivation. Because a modified progressive ratio procedure was used in this study, a break point-like analysis was performed to compare break point measures to the behavioral economics parameters of *Q*_0_ and α. Two measures were used in this analysis. One measure was the FR associated with a consumption of 0, representing the FR where none of the trials was completed; this measure is most similar to the traditional concept of break point and will therefore be called “break point” for this paper. The second measure was the smallest FR associated with at least one incomplete trial (First Failure Point). A Kaplan-Meier estimator was used because this analysis can utilize rats that did not reach their break point within our FR range (“censored” values; Rich et al., 2010).

### Statistics

Data are expressed as mean values ± 1 standard error of the mean. Statistics were performed using GraphPad Prism for Mac (Version 6, GraphPad Software Inc., La Jolla, CA) with an α level of 0.05. Fitting the Exponential Demand Equation and the Exponentiated Demand Equation to the data was performed with GraphPad Prism for Mac. Goodness of fit was determined by *r*^2^ value for each demand curve and all demand curve fits in the present study met the established criterion of *r*^2^ ≥ 0.30. An extra sum-of-squares F test was used to determine significant differences in *Q*_0_ and α for both demand equations. Correlations between *Q*_0_ and α were evaluated using a Pearson correlation (GraphPad Prism for Mac). Group differences in break point and first failure point (Kaplan-Meier estimator) were evaluated statistically using a log-rank test (GraphPad Prism for Mac).

## Results

### Demand characteristics of shock cessation in SD rats

#### Exponential demand equation analysis

**Figure 2:**
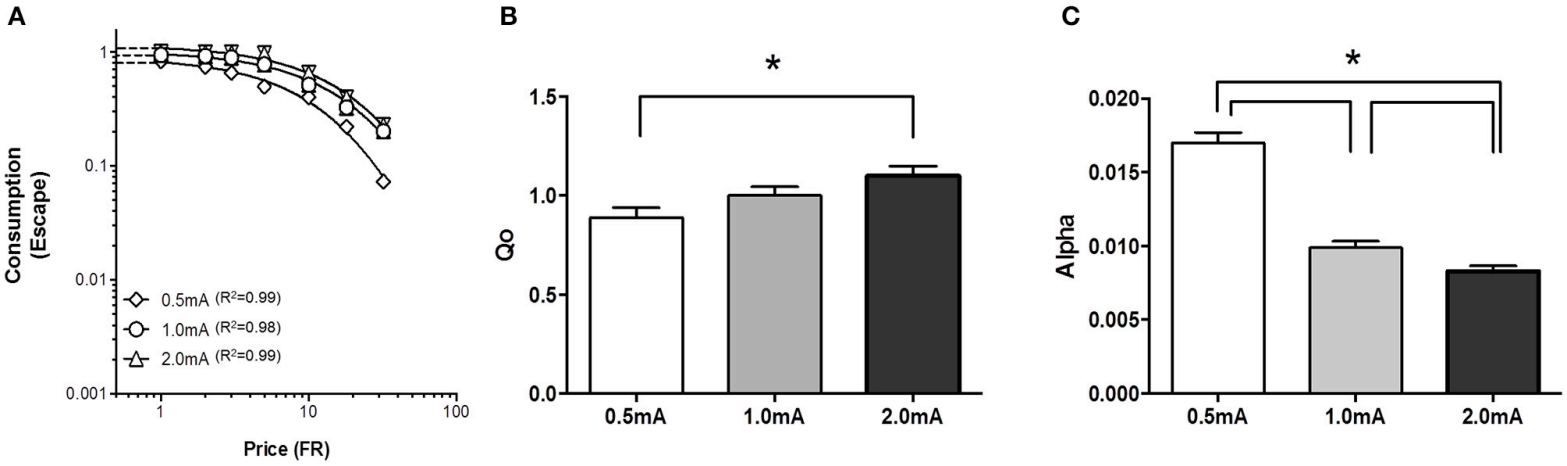
**Relief and motivation increased as footshock intensity increased**. Demand for negative reinforcement was assessed in Sprague Dawley (SD) rats (*n* = 13) at 3 different shock intensities. **(A)** A composite demand curve was generated by fitting the exponential demand equation to group means at each shock intensity. The symbols represent successful escapes and the lines represent the best fit demand curves for each shock intensity (diamonds = 0.5 mA, circles = 1.0 mA, and triangles = 2.0 mA). **(B)** The innate reinforcing value of footshock (*Q*_0_) significantly increased as shock intensity increased with a significant difference observed between 0.5 and 2.0 mA. **(C)** Motivation to escape shock (α) significantly increased (lower α) as shock intensity increased. α significantly differed between 0.5 and 1.0 mA, 0.5 and 2.0 mA, and 1.0 and 2.0 mA. ^*^p < 0.05.

Our novel procedure was initially tested in outbred SD rats to assess whether behavior economic parameters were sensitive to shock intensity. A demand curve for shock cessation (negative reinforcement) was established for each SD rat (*n* = 13) at 0.5, 1.0, and 2.0 mA footshock (Figure 2A). Performance at each shock intensity was stable; log_10_(α) values of the 3 sessions at each intensity were within 15% of the mean those days. *Q*_0_ of negative reinforcement was significantly greater with high shock current than with low shock current, suggesting escape from high shock current was more relieving with greater hedonic value than low shock current (Figure 2B) [*F*_(2, 15)_ = 6.7, *p* < 0.05]. Further, analysis revealed that *Q*_0_ was significantly greater at 2.0 mA compared to 0.5 mA [*F*_(1, 10)_ = 13.0 *p* < 0.05], but did not differ between 0.5 and 1.0 mA [*F*_(1, 10)_ = 3.3] or 1.0 and 2.0 mA [*F*_(1, 10)_ = 3.6]. α decreased as shock intensity increased, suggesting rats were more motivated to escape high shock current than low current (Figure 2C) [*F*_(2, 15)_ = 90, *p* < 0.001]. *Post-hoc* analysis revealed that α significantly decreased between 0.5 and 1.0 mA [*F*_(1, 10)_ = 90, *p* < 0.001], 0.5 and 2.0 mA [*F*_(1, 10)_ = 163, *p* < 0.001] and between 1.0 and 2.0 mA [*F*_(1, 10)_ = 7.6, *p* = 0.022]. *r*^2^ values for demand curves were between 0.99 and 0.98.

**Figure 3:**
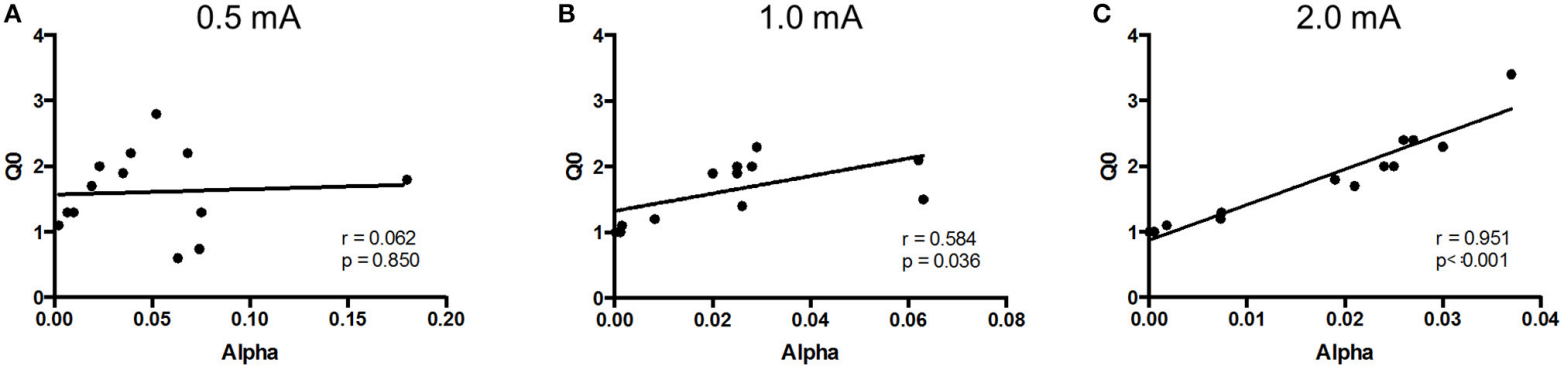
**Relief (*Q*_0_) and motivation (α) were positively correlated at high shock intensities**. The relationship between *Q*_0_ and α was assessed in Sprague Dawley (SD) rats (*n* = 13) at each shock intensity. **(A)** At 0.5 mA, relief experienced from shock cessation was uncorrelated to motivation. **(B,C)** However, at 1.0 mA **(B)** and 2.0 mA **(C)**, rats that experienced greater relief from shock cessation showed greater motivation.

The relation between *Q*_*o*_ and α was assessed for negative reinforcement at each of the three different shock intensities. Pearson correlation analysis revealed a positive relation between hedonic value and motivation at high, but not low, shock intensity (Figure 3). *Q*_*o*_ and α were significantly correlated at 1.0 mA (*r* = 0.584, *p* = 0.036) and 2.0 mA (*r* = 0.951, *p* < 0.001.), but not at 0.5 mA (*r* = 0.062, *p* > 0.1). Thus, SD rats modulated motivation to the hedonic value of negative reinforcement, but only at higher shock intensities.

#### Exponentiated demand equation analysis

**Figure 4:**
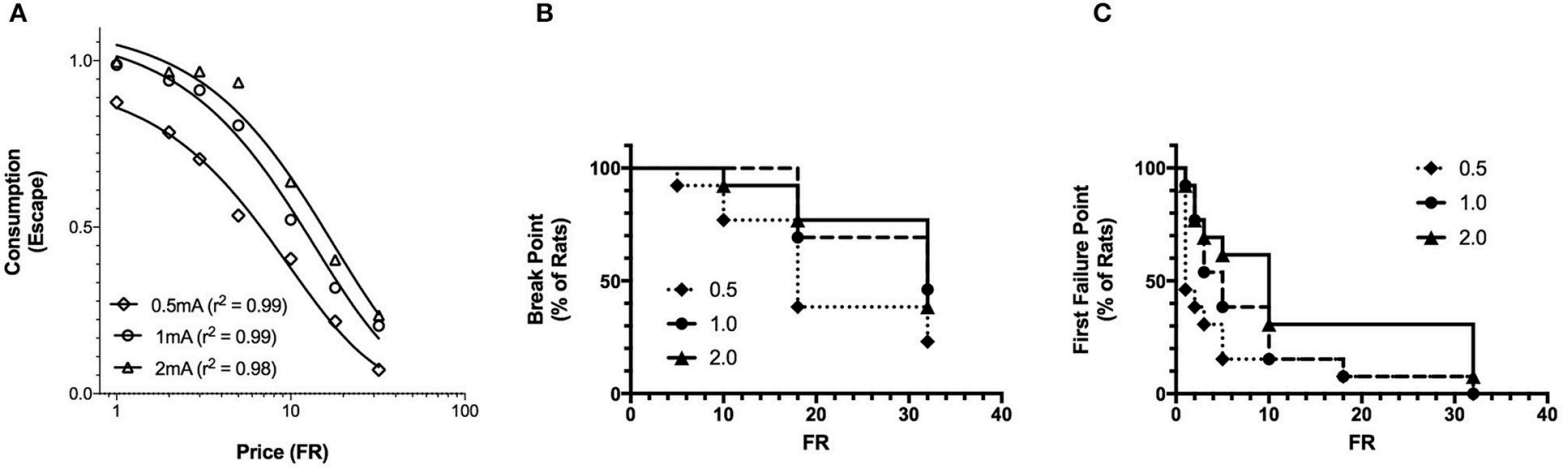
**Exponentiated and break point analysis of increasing footshock intensity. (A)** A composite demand curve was generated by fitting the exponentiated demand equation (Equation 2) to mean consumption at each shock intensity. The symbols represent successful escapes and the lines represent the best fit demand curves for each shock intensity (diamonds = 0.5 mA, circles = 1.0 mA, and triangles = 2.0 mA). *Q*_0_ at 1.0 and 2.0 mA differed from 0.5 mA, but did not differ from each other. α differed at all 3 stimulus intensities. The fits (*r*^2^) of the exponentiated and exponential demand equation were similar. **(B)** For each FR, the proportion of rats reaching their break point is represented by a reduction in the survival curve (survival is equivalent to the rats completing at least one trial for the FR). Differences in break point were not observed between shock intensities. **(C)** For each FR, the proportion of rats reaching their first failure point is represented by a reduction in the survival curve (survival equals rats successfully completing all trials for the FR). The first failure point was different across shock intensities, significantly differing between 0.5 and 2.0 mA, but not elsewhere.

The basic form of the exponentiated demand curve (Figure 4A) differs from the exponential demand curve (Figure 2A) because the exponentiated demand analysis uses a linear y-axis while the exponential demand curve is plotted on a logarithmic y-axis. The fit of the exponential and exponentiated equations to the data were similar (Table 1). Using the exponentiated equation, *Q*_0_ was significantly greater for high shock intensity than for low shock intensity [*F*_(2, 15)_ = 5.2, *p* = 0.019]. *Q*_0_ at 1.0 and 2.0 mA shock intensities was significantly greater than *Q*_0_ at 0.5 mA [0.5 vs. 1 mA: *F*_(1, 10)_ = 11, *p* = 0.007; 0.5 vs. 2.0 mA: *F*_(1, 10)_ = 8.8, *p* = 0.014]. *Q*_0_ at 1.0 and 2.0 mA did not differ [*F*_(1, 10)_ = 0.16]. α differed between the three shock intensities [*F*_(2, 15)_ = 23, *p* < 0.001]. α significant decreased between 0.5 and 1.0 mA [*F*_(1, 10)_ = 32, *p* < 0.001], 0.5 and 2.0 mA [*F*_(1, 10)_ = 42, *p* < 0.001], and between 1.0 and 2.0 mA [*F*_(1, 10)_ = 5.6, *p* = 0.04]. *r*^2^ values for exponentiated demand curves ranged between 0.98 and 0.99.

**Table 1:** **Comparison of exponential equation and exponentiated equation analyses**.

**SD - Stimulus Intensity**	**Exponential equation**				**Exponentiated equation**			
	**0.5 mA**	**1 mA**		**2 mA**	**0.5 mA**	**1 mA**		**2 mA**
*Q*_0_	0.89 ± 0.05	1.0 ± 0.05		1.1 ± 0.05	0.95 ± 0.03	1.1 ± 0.03		1.1 ± 0.04
α (× 10^−3^)	18.0 ± 0.73	10.0 ± 0.45		8.7 ± 0.37	18.0 ± 1.0	11.0 ± 0.69		8.8 ± 0.8
*r*^2^	0.99	0.98		0.99	0.99	0.99		0.98
**SD vs. WKY Aversive**	**SD**		**WKY**		**SD**		**WKY**	
*Q*_0_	1.2 ± 0.07		1.1 ± 0.14		1.1 ± 0.04		1.1 ± 0.07	
α (× 10^−3^)	51.0 ± 4.6		21.0 ± 4.8		24.0 ± 2.1		7.9 ± 2.0	
*r*^2^	0.99		0.84		0.98		0.82	
**SD vs. WKY Appetitive**	**SD**		**WKY**		**SD**		**WKY**	
*Q*_0_	1.3 ± 0.15		1.4 ± 0.10		1.1 ± 0.08		1.2 ± 0.09	
α (× 10^−3^)	2.7 ± 0.22		6.1 ± 0.32		1.6 ± 0.3		5.0 ± 0.61	
*r*^2^	0.95		0.99		0.9		0.97	

In summary, *Q*_0_ and α changed as a function of footshock intensity. Escape from high shock currents offered more relief (greater *Q*_0_) than escape at low shock currents. Motivation to escape (lower α) was also greater at high shock currents compared to low shock currents. Fits of the exponential and exponentiated demand equations to data were remarkably similar.

#### Break point analyses

The effects of shock intensity on break point and first failure point were investigated as additional measures of motivation. The FR where 50% of the rats reached their break point (median FR) was FR-18, FR-32, and FR-32 for 0.5, 1.0, and 2.0 mA shock intensities, respectively (Figure 4B). Break point did not differ between shock intensities [$\chi_{\left(2\right)}^{2}$ = 3.624]. In contrast, First Failure Point differed between the shock intensities [$\chi_{\left(2\right)}^{2}$ = 6.53, *p* = 0.038] (Figure 4C). The median FR for First Failure Point was FR-1, FR-5, and FR-10 for 0.5, 1.0, and 2.0 mA, respectively. First Failure Point was significantly greater at 2.0 mA compared to 0.5 mA [$\chi_{\left(1\right)}^{2}$ = 5.299, *p* = 0.021]. All other comparisons did not reach significance [$\chi_{\left(1\right)}^{2}$ <2.03]. Thus, break point was less sensitive than first failure point in distinguishing between motivation to escape different shock intensities. Moreover, first failure point was less sensitive than the behavioral economics parameter α in detecting differences in motivation between each of the three shock intensities.

### Demand characteristics of negative and positive reinforcement in anxiety vulnerable rats

To demonstrate the utility of our novel procedure for psychiatric disorders, we compared anxiety vulnerable WKY rats to outbred SD rats. WKY rats exhibit a behaviorally inhibited temperament and an exaggerated response to stress (Paré, 1989, 1994; Will et al., 2003; Servatius et al., 2008; McAuley et al., 2009). WKY rats also develop perseverative avoidance (Servatius et al., 2008; Beck et al., 2011; Jiao et al., 2011), a core symptom of anxiety disorders (American Psychiatric Association, 2013).

#### Negative reinforcement—exponential demand equation

**Figure 5:**
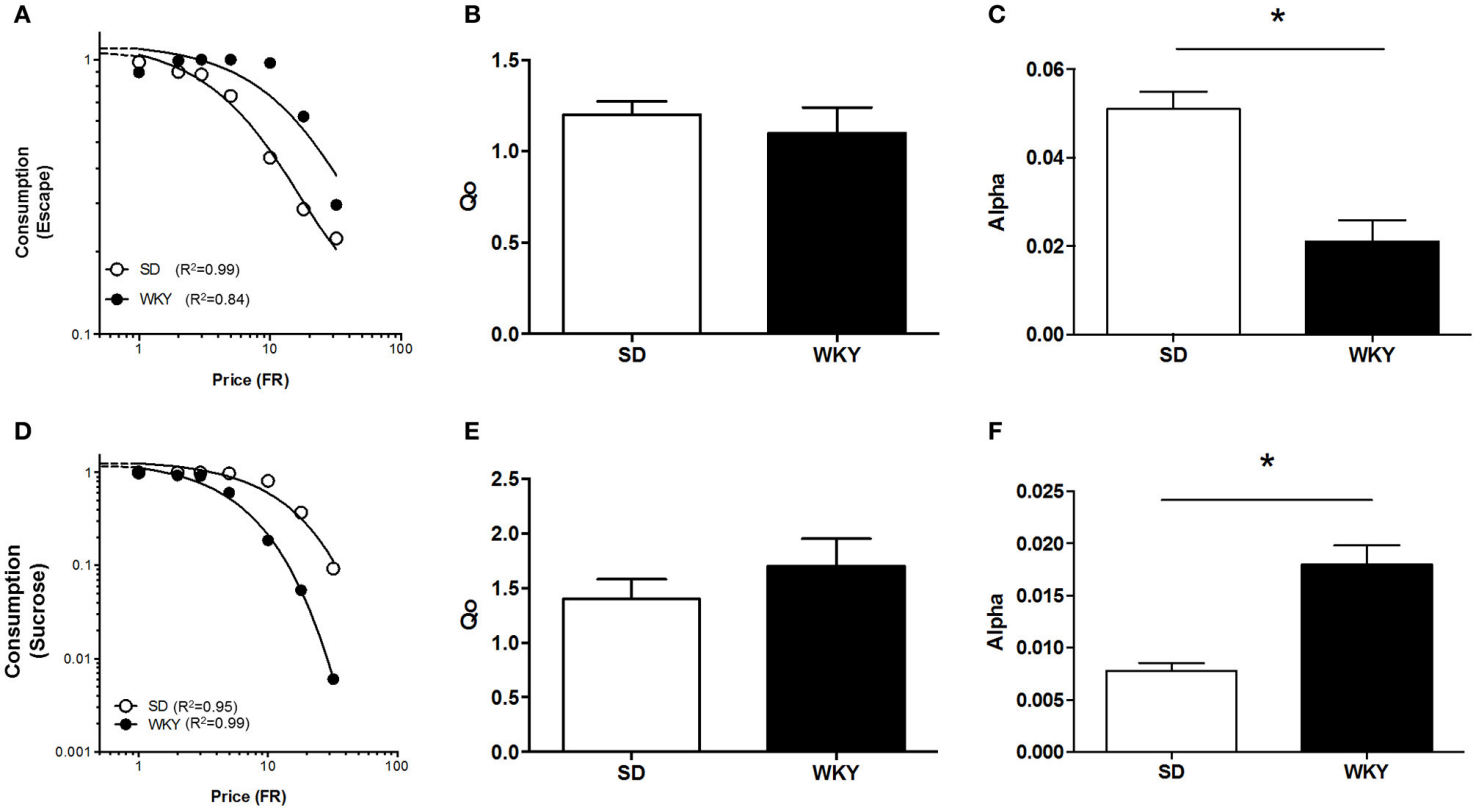
**Differential motivation for positive and negative reinforcement in anxiety vulnerable WKY rats**. The demand for shock cessation and sucrose were compared between Sprague Dawley (SD) and anxiety vulnerable Wistar Kyoto (WKY) rats. **(A)** Composite demand curves for shock cessation were generated from the exponential demand equation for group means of SD (*n* = 8) and WKY (*n* = 6) rats. The symbols represent successful escapes made for a given FR and lines represent best fit demand curves (open circles = SD rats, closed circles = WKY rats). **(B)** *Q*_0_ did not differ between SD and WKY rats. **(C)** WKY rats were more motivated to escape footshock (smaller α) compared to SD rats. **(D)** Composite demand curves for sucrose were generated from the exponential demand equation for group means of SD (*n* = 11) and WKY rats (*n* = 11). Symbols represent successful trials in which sucrose was obtained at a given FR and lines represent best fit demand curves (open circles = SD rats, closed circles = WKY rats). **(E)** *Q*_0_ did not differ between SD and WKY rats. **(F)** WKY rats were less motivated to obtain sucrose (greater α) than SD rats. ^*^p < 0.05.

Demand characteristics for shock cessation were determined for SD (*n* = 8) and WKY (*n* = 6) rats at 1.0 mA footshock (Figure 5A). *Q*_0_ did not differ between SD and WKY rats, suggesting that shock cessation offered equivalent relief to both strains (Figure 5B) [*F*_(1, 10)_ = 0.018]. α was significantly lower in WKY rats compared to SD rats, providing evidence for increased motivation in WKY rats to escape shock (Figure 5C) [*F*_(1, 10)_ = 17, *p* < 0.001].

**Figure 6:**
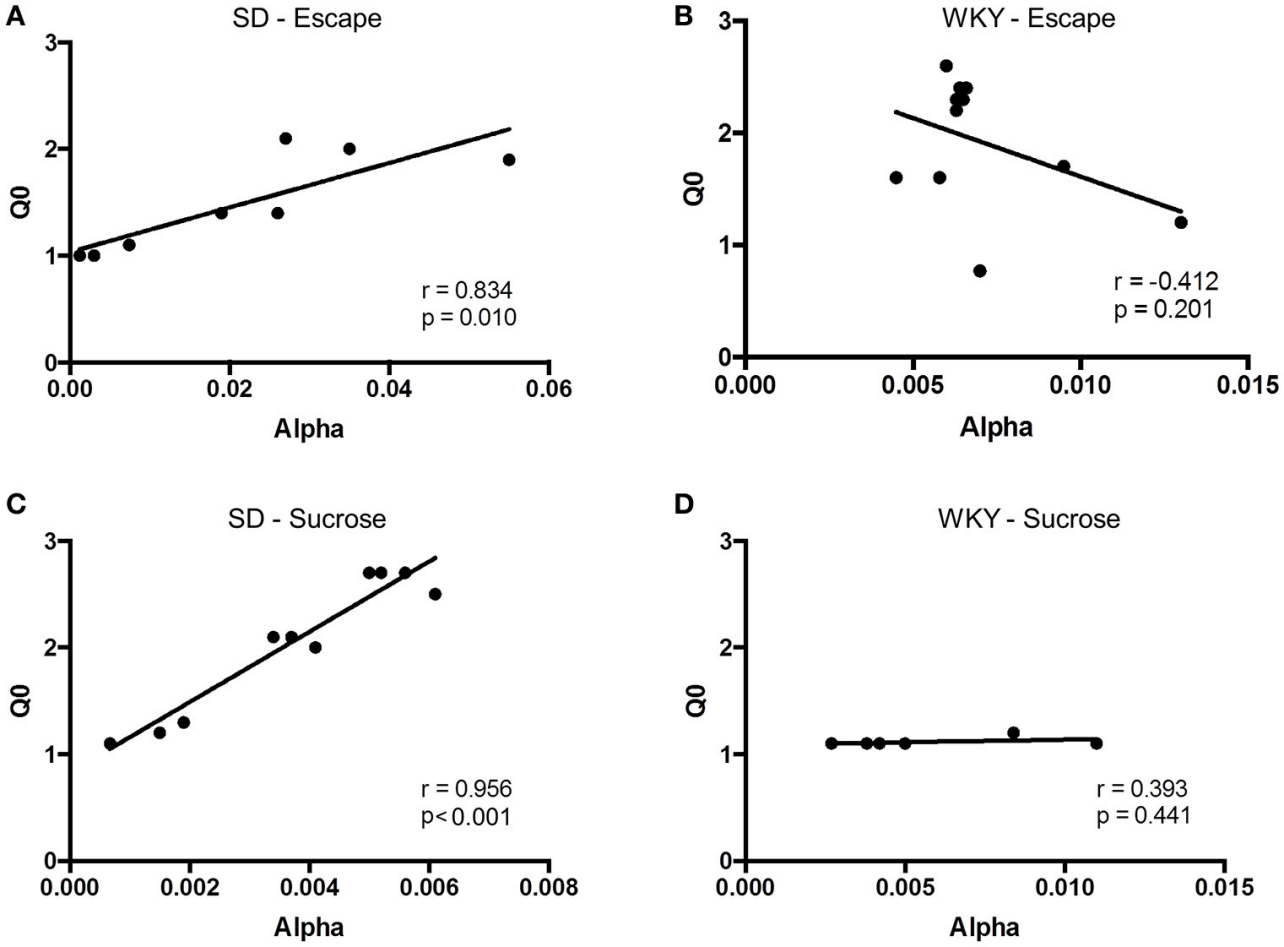
**Relationship between relief (*Q*_0_) and motivation (α) differ between strains**. The relationship between *Q*_0_ and α was assessed in Sprague Dawley (SD) and Wistar Kyoto (WKY) rats for negative and positive reinforcement. **(A)** In SD rats (*n* = 8), relief was positively correlated with motivation, such that rats showing greatest relief from shock cessation also demonstrated the most motivation to escape. **(B)** In contrast to SD rats, WKY rats (*n* = 6) showed no correlation between relief and motivation for negative reinforcement. **(C)** Similar to negative reinforcement, a positive correlation was observed in SD rats (*n* = 11) between pleasurability from sucrose and motivation to obtain sucrose. **(D)** Hedonic value and motivation for sucrose was not observed in WKY rats (*n* = 11).

Hedonic value and motivation were positively correlated in SD but not WKY rats. For SD rats, *Q*_*o*_ and α were significantly correlated at 1 mA footshock (Figure 6A; *r* = 0.834, *p* = 0.01). In contrast, *Q*_*o*_ and α were not correlated for WKY rats (Figure 6B; *r* = −0.412, *p* > 0.1).

#### Negative reinforcement—exponentiated demand equation

**Figure 7:**
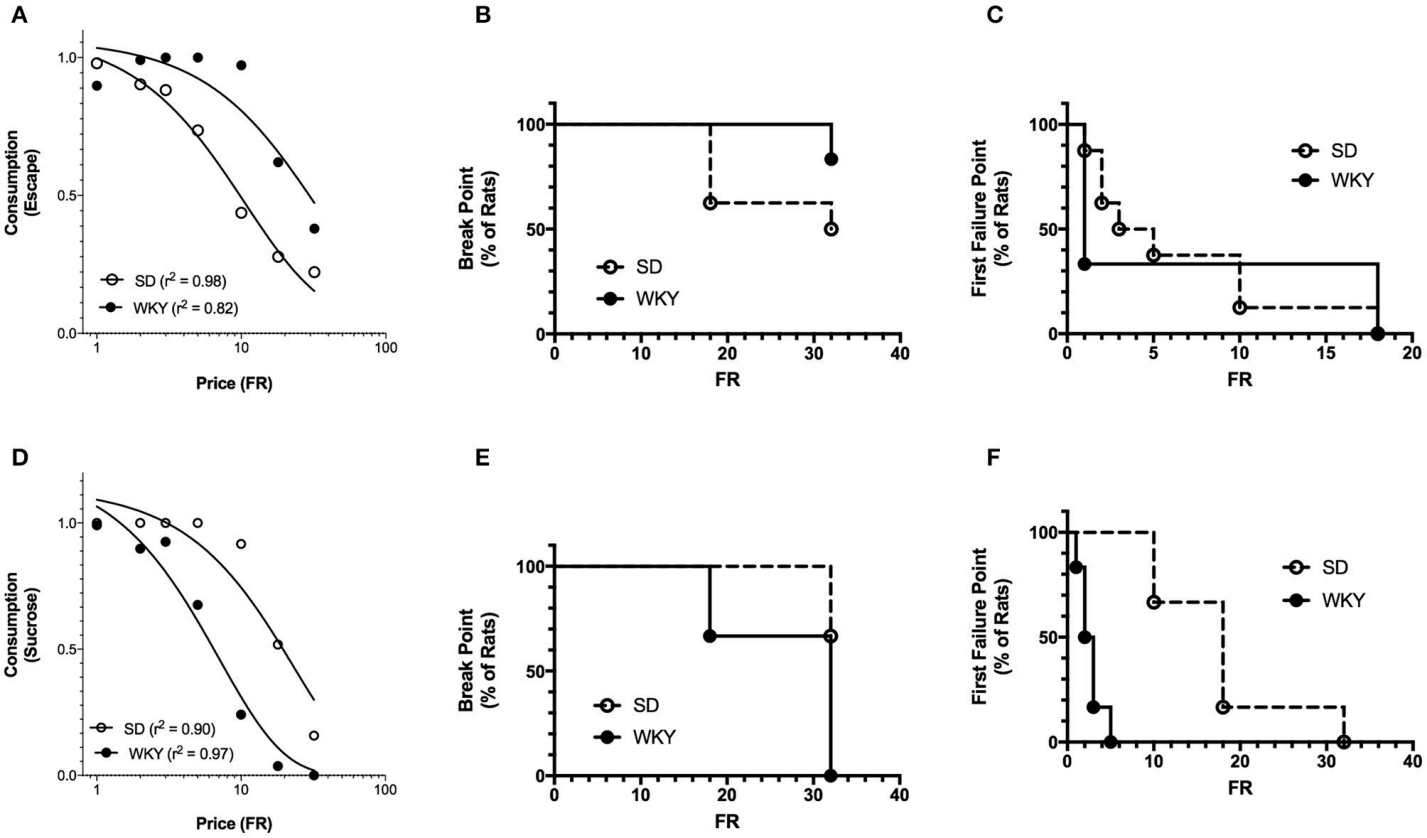
**Exponentiated and break point analysis of positive and negative reinforcement in SD and WKY rats. (A)** Demand curves were generated by fitting the exponentiated demand equation to mean consumption for Sprague Dawley (SD) and Wistar Kyoto (WKY) rats. Symbols represent successful escapes and the lines represent the best fit demand curves for each strain (open circle = SD rats, closed circle = WKY rats). *Q*_0_ did not differ between SD and WKY rats, whereas motivation was greater (lower α) for WKY rats as compared to SD rats at 1.0 mA shock intensity. Neither break point **(B)** nor first failure point **(C)** differed between strains. **(D)** Demand curves were generated by fitting the exponentiated demand equation to mean consumption for SD and WKY rats. Symbols represent sucrose pellets received and the lines represent the best fit demand curves for each strain (open circle = SD rats, closed circle = WKY rats). *Q*_0_ did not differ between SD and WKY rats, but motivation was less (higher α) for WKY rats as compared to SD rats. WKY rats had significantly smaller break points **(E)** and first failure points **(F)** than SD rats.

The demand characteristics of shock cessation for SD and WKY rats were also analyzed using the exponentiated equation (Figure 7A). Fit of the exponential and exponentiated equations to the data were similar (Table 1). Both equations fit the data from SD rats slightly better than WKY rats, although fits for both rat strains were excellent (*r*^2^ = 0.82–0.99). The extrapolated results from the exponentiated equation analysis were similar to those obtained from the exponential demand equation (Table 1). *Q*_0_ did not differ between SD and WKY rats in escaping from 1.0 mA footshock [*F*_(1, 10)_ = 0.07]. α was significantly lower in WKY rats compared to SD rats, suggesting WKY rats were more motivated to escape footshock than SD rats [*F*_(1, 10)_ = 21, *p* = 0.001].

#### Negative reinforcement—break point analyses

SD and WKY rats did not differ on break point [$\chi_{\left(1\right)}^{2}$ = 1.775] (Figure 7B) or first failure point [$\chi_{\left(1\right)}^{2}$ = 0] (Figure 7C).

#### Positive reinforcement—exponential demand equation

A demand curve was established for positive reinforcement for SD (*n* = 11) and WKY (*n* = 11) rats using sucrose pellets (Figure 5D). *Q*_0_ did not differ between SD and WKY rats, suggesting similar hedonic set points for sucrose (Figure 5E) [*F*_(1, 10)_ = 0.098]. In contrast to negative reinforcement, α was significantly higher in WKY rats compared to SD rats, evidence that motivation for consuming sucrose was less for WKY rats compared to SD rats (Figure 5F) [*F*_(1, 10)_ = 69, *p* < 0.001].

Hedonic value for sucrose and motivation to obtain sucrose were positively correlated for SD but not WKY rats. For SD rats, *Q*_*o*_ and α were significantly and positively correlated (Figure 6C; *r* = 0.956, *p* < 0.001). However, these two measures were not correlated in WKY rats (Figure 6D; *r* = 0.393, *p* > 0.1).

#### Positive reinforcement—exponentiated demand equation

The fit of the exponentiated equation was similar to that of the exponential demand equation, and excellent for data from SD and WKY rats (Figure 7D, Table 1). For the exponentiated demand analysis, *Q*_0_ did not differ between SD and WKY rats (Table 1) [*F*_(1, 10)_ = 0.51]. In contrast, α was higher for WKY rats compared to SD rats, providing evidence of reduced motivation for consuming sucrose in WKY rats [*F*_(1, 10)_ = 30, *p* < 0.001].

#### Positive reinforcement—break point analyses

Break point was significantly different between SD and WKY rats for positive reinforcement (Figure 7E). Median FR for break point was greater than FR-32 for SD rats and equal to FR-32 for WKY rats. The lower break point for WKY rats was significant as compared to SD [$\chi_{\left(1\right)}^{2}$ = 6.176, *p* = 0.013]. First Failure Point was also significantly different between SD and WKY rats for positive reinforcement [$\chi_{\left(1\right)}^{2}$ = 11.84, *p* < 0.001] (Figure 7F). Median FR for SD and WKY rats were FR-18 and FR-2.5, respectively, demonstrating reduced motivation for positive reinforcement in WKY rats as compared to SD rats.

In summary, shock cessation offered equivalent relief between SD and WKY rats. Sucrose was also similarly reinforcing between SD and WKY rats. Although hedonic value (*Q*_0_) of positive and negative reinforcement did not differ between strains, motivation (α) to obtain these reinforcements differed. WKY rats were *more* motivated to escape shock compared to SD rats. This conclusion was supported by analyses using the both exponential and exponentiated demand equations. In contrast, WKY rats were *less* motivated to obtain sucrose than SD rats, and this conclusion was supported by the two demand curve analyses, as well as break point and first failure point analyses. Additionally, hedonic value and motivation were positively correlated in SD rats for both positive and negative reinforcement. In contrast, hedonic value and motivation were not correlated in WKY rats for either type of reinforcement. The contrasting results between demand for shock cessation and sucrose emphasize the importance of assessing appetitive and aversive motivated behaviors, and parallels the negative bias for attention observed in individuals with anxiety disorders. Moreover, the lack of correlation of hedonic value and motivation for WKY rats suggest that anxiety vulnerability may be associated with an inability to adjust motivation to an appropriate level of hedonic value.

## Discussion

In this study, we describe a novel procedure to characterize the hedonic and motivational components of negative reinforcement using behavioral economics. We propose that *Q*_0_ is a measure of the hedonic component of relief following termination of a noxious event, whereas α is an index of demand elasticity or motivation to terminate a noxious event. In SD rats, footshock cessation was more relieving at high shock intensity than low shock intensity (increasing *Q*_0_). SD rats also displayed more motivation to escape high intensity footshock compared to low intensity shock. In a second study, we demonstrate the utility of a behavioral economic approach to the study of mental health disorders by applying our procedure to an animal model of behaviorally inhibited temperament, a known vulnerability for anxiety disorders (Kagan et al., 1987, 1989; Rosenbaum et al., 1993; Schwartz et al., 1999). Shock cessation offered SD and WKY rats equivalent relief, but WKY rats demonstrated less elasticity, suggesting they were more motivated to escape shock compared to SD rats. In contrast, WKY rats showed greater elasticity to obtain sucrose, suggesting they were less motivated to consume sucrose compared to SD rats. The relative differences in motivation for positive and negative reinforcement between the strains occurred in spite of similar hedonic values between strains; thus, hedonic and motivational components are independent for both positive and negative reinforcement (Hursh and Silberberg, 2008; Bentzley et al., 2013). These results demonstrate the utility of a behavioral economic approach to investigate hedonic assessment and motivation of negative reinforcement, which is critical to the understanding of anxiety disorders.

The concepts of sensing pain and pain relief are closely related and difficult to tease apart. We interpret our data as resulting from pain relief due to cessation of shock, and separate pain relief into hedonic and motivational components. One question is whether our results could be equally interpreted as due to the sensation of pain rather than pain relief. Our interpretations are based on the finding that signals predicting the onset of shock are associated with fear, whereas signals associated with shock cessation (i.e., pain relief) are reinforcing (Wiech and Tracey, 2013; Navratilova and Porreca, 2014). Moreover, termination of tail pinch is associated with increased dopamine release in NAc, whereas the tail pinch itself is associated with suppression of extracellular dopamine in the NAc (Park et al., 2015). In the present study, escape response latency decreased during acquisition leading to reduction of the number of shocks received; this pattern is consistent with a reinforced response. Furthermore, pain sensitivity is not different between SD and WKY rats (unpublished results using threshold for vocalization and flinch). Based on these results, might *Q*_0_ be related to pain sensitivity, while α is related to pain relief (shock cessation)? While this idea is possible, it would be inconsistent with the separation of reinforcement into hedonic and motivational components that is paramount to behavioral economics and current views of reinforcement (Hursh and Silberberg, 2008; Berridge et al., 2009; Salamone and Correa, 2012).

α values obtained with 1 mA footshock in SD rats varied in the two studies (shock intensity and strain comparison). There are several possibilities that may explain this discrepancy. First, SD rats were naïve at the start of testing in the strain comparison, whereas rats in the shock intensity study were tested at 0.5 mA prior to testing at 1 mA. This prior experience with a lower shock intensity and/or familiarity with the demand procedure may be responsible for differences in α. Additionally, as a result of the repeated measures design for the shock intensity study, rats were exposed to a greater number of testing sessions than in the strain comparison study. The increase in demand is reminiscent of escalation observed in drug self-administration studies. Long-access models of drug addiction, which give rats extended access to a particular drug, increase Qo and decrease α (Bentzley et al., 2014). It is possible that prolonged exposure to negative reinforcement alters demand parameters much in the same way long-access models alter demand parameters for self-administration studies. These differences do not preclude the conclusion that SD and WKY rats differ in motivation because both strains were treated similarly within the study, but they do caution against comparing measures across the two studies.

The exponential demand equation provides a well-established method for separating the hedonic and motivational properties of a reinforcer (Hursh and Silberberg, 2008; Bentzley et al., 2013), a concept reminiscent of *liking* and *wanting* components of positive reinforcement outlined by the incentive salience theory (Berridge and Robinson, 1998). *Liking* refers to the innate pleasurability immediately gained from the reinforcer. *Liking* can be objectively measured by affective facial expressions (Fox and Davidson, 1986; Rosenstein and Oster, 1988; Berridge et al., 1989; Berridge, 2000; Steiner et al., 2001; Pecina et al., 2003). In contrast, *wanting* is an intense motivation to obtain a reinforcer. *Wanting*, as used here, is distinct from a cortical type of wanting, which uses explicate goals, is conscious and can be rationalized; instead, it is a type of subcortical wanting that is not always rational (Dayan and Balleine, 2002; Pecina et al., 2003; Daw et al., 2005; Tindell et al., 2005; Faure et al., 2008; Robinson and Berridge, 2008; Berridge, 2009). In drug addiction, the *wanting* system becomes selectively amplified such that *wanting* becomes pathological and can be sought out even if *liking* is low (Robinson and Berridge, 2008).

The behavioral economics parameters *Q*_0_ and α are analogous to the “liking” and “wanting” components of positive reinforcers discussed above. *Q*_0_ refers to an individual's hedonic set point that describes the amount of a reinforcer an individual would consume when no work is required (Hursh and Silberberg, 2008; Bentzley et al., 2013). α is a measure of demand elasticity, which is inversely related to motivation (Hursh and Silberberg, 2008; Bentzley et al., 2013). Like the incentive salience theory, *Q*_0_ and α are distinguishable independent components of a reinforcer. For example, *Q*_0_ is significantly greater for saccharin than food, but rats are more motivated to work for food than saccharin, suggesting that while rats preferred the sweet taste of saccharin, they are driven to consume food because it is a basic necessity (Bentzley et al., 2013). Cocaine addiction provides another example of the independence of *Q*_0_ and α (Bentzley et al., 2014). In rats self-administering cocaine, α and *Q*_0_ are uncorrelated, indicating that rats with a greater demand for cocaine do not necessarily have a greater preference for cocaine (Bentzley et al., 2014). Thus, the demand parameters *Q*_0_ and α provide independent measures of hedonic and motivational aspects of a reinforcer, respectively.

Differences exist in the interpretation of the demand parameters for positive and negative reinforcement, specifically when discussing *Q*_0_. In the present study, we propose *Q*_0_ is a measure of relief. As previously described, *Q*_0_ is used as a measure of hedonic set point when assessing positive reinforcers. In the traditional behavioral economic approach, *Q*_0_ for positive reinforcement is greatly influenced by satiety. However, this is not likely the case with negative reinforcement. If given the opportunity, it would be unlikely that anyone would forgo the opportunity to escape an aversive event. Still we would expect individual differences in perceived relief. The literature on chronic pain has shown that relief from pain is rewarding, and relief experienced from the cessation of pain will differ among individuals, just as pain threshold differ (Wiech and Tracey, 2013; Navratilova and Porreca, 2014). We demonstrated that relief from shock cessation did not differ between SD and WKY rats, even though motivation to escape shock differed. It is possible that the lack of strain differences in *Q*_0_ is due to a ceiling effect, as no differences were observed for *Q*_0_ associated with 1 and 2 mA in SD rats. Future studies should examine WKY rats at 0.5 mA, which was associated with a significantly lower *Q*_0_ than that at 1 or 2 mA in SD rats. Interestingly, results with positive reinforcement were similar to those resulting from negative reinforcement; that is, the hedonic value of sucrose did not differ between SD and WKY rats, even though motivation to consume sucrose differed. These results suggest that the hedonic value and motivation are independent qualities of negative reinforcement, consistent with reports showing motivation for positive reinforcement is independent of hedonic set point or preference.

The procedure described in the present study is not one traditionally used in behavioral economics. The typical measure of behavioral economics is the amount of consumption (typically food or drugs) that occurs in a set time period (Christensen et al., 2008a; Oleson and Roberts, 2009; España et al., 2010; Bentzley et al., 2013). Because of ethical concerns, we used a trial based procedure that limited the maximum shocks an animal could receive on each trial (20 maximum) if they failed to complete the FR requirements. Additionally, this procedural design gave rats a break between shock periods (intertrial interval). The term consumption is a difficult one to relate to negative reinforcement. In our procedure, we used completed FR trials as a measure of consumption, analogous to the number of reinforcements received for completing the FR in a traditional behavioral economics procedure. The form of the demand curve and high goodness of fit of the exponential demand equation to our data suggest that our novel procedure for negative reinforcement is appropriate for the well-described behavioral economics analysis used to characterize positive reinforcement (Christensen et al., 2008a; Hursh and Silberberg, 2008; Bentzley et al., 2013).

As a further test of our novel procedure, the data were analyzed using an exponentiated form of the demand equation. While the Hursh and Silberberg exponential demand equation (Equation 1) provides excellent fits to data from a wide variety of studies, one drawback is the inability to use 0 values of consumption. In the present study, 0 values of the consumption ratio were replaced with 1/10 the value of the lowest non-zero value, an accepted though not universal practice in using the exponential demand equation (Koffarnus et al., 2015). However, a recent study found that replacement of 0 values with non-zero values can alter *Q*_0_ and α, and suggested that using an exponentiated equation provides a more accurate method because it can utilize true 0 values of consumption data (Koffarnus et al., 2015). In the present study, both exponential and exponentiated equations provided excellent fits to our data. Conclusions reached regarding hedonic value and motivation as a function of stimulus intensity and strain differences were similar when calculated from these equations. Thus, our novel procedure provides data that conform well to two models used to characterize behavioral economics in a variety of situations.

Break point is another measure of motivation used in progressive ratio procedures. In the progressive ratio task, the number of required responses to complete the trial continues to increase in some pre-determined manner until the animal fails to complete the response requirement. Break point is the FR on which the animal stops responding for a set amount of time. Since our procedure is not a true progressive ratio task, we could not use a traditional measure of break point. Instead, we used two measures to provide an analogous type of analysis. One measure was the FR on which a rat did not receive any reinforcement (i.e., failed to complete all trials); this measure is most similar to the traditional measure of breakpoint. The second measure was the FR in which a rat first failed to complete a trial (first failure point). A Kaplan-Meier estimate was used to analyze these measures because this analysis can accommodate rats that failed to reach their break point (or first failure point) in the range of FRs used in the present study (“censured” values). While the Kaplan-Meier analysis of break point and first failure point described significant differences in motivation between SD and WKY rats, this analysis was generally less sensitive in finding motivational differences than α values from the exponential or exponentiated demand equations. The relative insensitivity of break point analyses, as compared to behavioral economics measures, may be due to the fact that our procedures were developed specifically for behavioral economics analyses, and a maximum FR of 32 was used whether or not a break point was reached in all animals. In summary, a break point or first failure point was less sensitive in measuring motivation than the behavior economic parameter of α, although this may be due to the experimental design. Even so, the behavioral economic analysis has the additional benefit of describing hedonic value of reinforcement.

The utility of a behavioral economics approach to study negative reinforcement in anxiety disorders was exemplified by the demand characteristics of SD and anxiety vulnerable WKY rats. Relief from shock cessation was similar between SD and anxiety vulnerable WKY rats. However, WKY rats were more motivated to escape shock than SD rats. Increased demand for shock cessation in anxiety vulnerable rats is analogous of the increased demand for drugs in substance abuse (Robinson and Berridge, 2008). It has been argued that motivation drives substance abuse and may serve as a biomarker for abuse severity. For example, demand (α) is associated with years of abuse for heroin and cocaine and intensity of cravings in alcoholics (Petry, 2001; MacKillop et al., 2010). Moreover, the incentive sensitization theory of drug abuse implicates amplification of the *wanting* system with addiction. Thus, escape and avoidant behavior in anxious individuals may be driven from an intense motivation to obtain negative reinforcement, rather than an enhanced relief experienced from escaping or avoiding an aversive event.

In the present study, negative reinforcement was separated into hedonic and motivational components. We propose the use of the terms *relief* and *survival* to describe these components, respectively. Here, survival refers to a drive for self-preservation. Obviously a drive to survive is advantageous, but if amplified in response to non-threating stimuli, the survival drive will become maladaptive. We propose the survival drive is amplified in anxiety vulnerable individuals and underlies the development of pathological avoidance. Thus, individuals with anxiety disorders may be more sensitive to viewing escape (and avoidance) as necessary for survival. Support for this idea comes from the finding that hedonic value and motivation were not correlated in WKY rats, as it was for SD rats. In this case, WKY rats are unable to optimally adjust motivation to correspond with appropriate hedonic value of the reinforcer, as SD rats did. Studies on pain and pain relief suggest that the nucleus accumbens and VTA may mediate the proposed *relief* and *survival* components of negative reinforcement, respectively (Andreatta et al., 2012; Huston et al., 2013; Wiech and Tracey, 2013; Navratilova and Porreca, 2014; Park et al., 2015). One possibility is that amplification of the survival drive develops from sensitization of the mesolimbic dopaminergic system, an idea similar to the incentive sensitization theory of drug addiction (Robinson and Berridge, 2008). Altogether, the concept of *relief* and *survival* components of negative reinforcement may better our understanding of pathological avoidance.

In summary, a novel procedure is described using behavioral economics analysis of negative reinforcement; this approach has unique utility for the study of stress-related psychopathology. In the present study, hedonic and motivational components of negative reinforcement were assessed for the first time. The hedonic component of relief from footshock cessation increased as shock intensity increased, and motivation to escape footshock increased as shock intensity increased. Our novel procedure was then applied to anxiety vulnerability where hedonic and motivational components of negative and positive reinforcement were compared in anxiety vulnerable WKY rats in comparison to outbred control SD rats. Anxiety vulnerability was associated with increased motivation to terminate footshock (survival), but was not different than the control strain for relief. Importantly, motivation to escape footshock was independent of the relief of footshock cessation, suggesting separate underlying mechanisms of survival motivation and relief. We argue that a risk factor for anxiety disorders may result from an amplification of the *survival* motivation component of negative reinforcement, and suggest further research on anxiety risk should focus on the motivational component of aversive stimuli.

## Author contributions

Study design and concept: JF and KP. Acquisition of data: JF. Analysis and interpretation of data: JF and KP. Preparation of manuscript: JF, KP, and KB. Obtained funding: KP and KB.

## Funding

This work was supported by the Biomedical Laboratory Research & Development and Department of Veterans Affairs Office of Research & Development grant I01BX000132 to KP, and the Graduate School of Biomedical Sciences, New Jersey Medical School–Rutgers Biomedical and Health Sciences.

### Conflict of interest statement

The authors declare that the research was conducted in the absence of any commercial or financial relationships that could be construed as a potential conflict of interest.
