# Corrigendum: Use of the Exponential and Exponentiated Demand Equations to Assess the Behavioral Economics of Negative Reinforcement

**DOI:** 10.3389/fnins.2017.00376

**Published:** 2017-06-30

**Authors:** Jennifer E. C. Fragale, Kevin D. Beck, Kevin C. H. Pang

**Affiliations:** ^1^Graduate School of Biomedical Sciences, Rutgers Biomedical and Health SciencesNewark, NJ, United States; ^2^Neurobehavioral Research Lab, Department of Veteran Affairs Medical Center–New Jersey Health Care SystemEast Orange, NJ, United States; ^3^Department of Pharmacology, Physiology and Neurosciences, New Jersey Medical School - Rutgers Biomedical and Health SciencesNewark, NJ, United States

**Keywords:** behavioral economics, anxiety disorders, exponential demand equation, motivation, anxiety, depression

In the original article, there was a mistake in the legends for **Figures 3, 6** as published. The correlations between *Q*_0_ and α were incorrectly interpreted. The correct legend appears below. The authors apologize for these errors and state that these errors do not change the scientific conclusions of the article in any way.

**FIGURE 3 |** *Q*_0_ and α were positively correlated at high shock intensities. The relationship between *Q*_0_ and α was assessed in Sprague Dawley (SD) rats (*n* = 13) at each shock intensity. **(A)** At 0.5 mA, *Q*_0_ and α were uncorrelated. **(B,C)** However, at 1.0 mA **(B)** and 2.0 mA **(C)**, rats that experienced greater relief from shock cessation (high *Q*_0_) showed reduced motivation (high α).

**FIGURE 6 |** Relationship between relief (*Q*_0_) and motivation (α) differed between strains. The relationship between *Q*_0_ and α was assessed in Sprague Dawley (SD) and Wistar Kyoto (WKY) rats for negative and positive reinforcement. **(A)** In SD rats (*n* = 8), *Q*_0_ was positively correlated with α, such that rats showing greatest relief from shock cessation (high *Q*_0_) demonstrated the least motivation to escape (high α). **(B)** In contrast to SD rats, WKY rats (*n* = 6) showed no correlation between relief and motivation for negative reinforcement. **(C)** Similar to negative reinforcement, a positive correlation was observed in SD rats (*n* = 11) between *Q*_0_ and α for sucrose. **(D)** A significant correlation of hedonic value and motivation for sucrose was not observed in WKY rats (*n* = 11).

In the original article, there was a mistake in the abstract. The correct abstract appears below.

Abnormal motivation and hedonic assessment of aversive stimuli are symptoms of anxiety and depression. Symptoms influenced by motivation and anhedonia predict treatment success or resistance. Therefore, a translational approach to the study of negatively motivated behaviors is needed. We describe a novel use of behavioral economics demand curve analysis to investigate negative reinforcement in animals that separates hedonic assessment of footshock termination (i.e., relief) from motivation to escape footshock. In outbred Sprague Dawley (SD) rats, relief increased as shock intensity increased. Likewise, motivation to escape footshock increased as shock intensity increased. To demonstrate the applicability to anxiety disorders, hedonic and motivational components of negative reinforcement were investigated in anxiety vulnerable Wistar Kyoto (WKY) rats. WKY rats demonstrated increased motivation for shock cessation with no difference in relief as compared to control SD rats, consistent with a negative bias for motivation in anxiety vulnerability. Moreover, motivation was correlated with relief in SD, but not in WKY. This study is the first to assess the hedonic and motivational components of negative reinforcement using behavioral economic analysis. This procedure can be used to investigate positive and negative reinforcement in humans and animals to gain a better understanding of the importance of motivated behavior in stress-related disorders.

**A correction has been made to Results, Demand Characteristics of Shock Cessation in SD rats: Exponential Demand Equation Analysis, Paragraph 2:**

The relation between *Q*_0_ and α was assessed for negative reinforcement at each of the three different shock intensities. Pearson correlation analysis revealed a positive relation between *Q*_0_ and α at high, but not low, shock intensity (**Figure 3**). *Q*_0_ and α were significantly correlated at 1.0 mA (*r* = 0.584, *p* = 0.036) and 2.0 mA (*r* = 0.951, *p* < 0.001.), but not at 0.5 mA (*r* = 0.062, *p* > 0.1). Thus, SD rats modulated motivation to the hedonic value of negative reinforcement, but only at higher shock intensities.

**The following corrections have been made to Results, Demand Characteristics of Negative and Positive Reinforcement in Anxiety Vulnerable Rats: Negative Reinforcement – Exponential Demand Equation, Paragraph 2:**

*Q*_0_ and α were positively correlated in SD but not WKY rats. For SD rats, *Q*_0_ and α were significantly correlated at 1mA footshock (**Figure 6A**; *r* = 0.834, *p* = 0.01). In contrast, *Q*_0_ and α were not correlated for WKY rats (**Figure 6B**; *r* = −0.412, *p* > 0.1).

Hedonic value for sucrose and motivation to obtain sucrose were correlated for SD but not WKY rats. For SD rats, *Q*_0_ and α were significantly and positively correlated (**Figure 6C**; *r* = 0.956, *p* < 0.001). However, these two measures were not correlated in WKY rats (**Figure 6D**; *r* = 0.393, *p* > 0.1).

The authors apologize for these errors and state that these errors do not change the main scientific conclusions of the article.

## Conflict of interest statement

The authors declare that the research was conducted in the absence of any commercial or financial relationships that could be construed as a potential conflict of interest.

